# Aspects of spermatogenesis in immature and mature specimens of the long-lived Greenland shark: Novelties concerning the germinal compartment’s assembly, complement of Sertoli cells and demise

**DOI:** 10.1371/journal.pone.0304475

**Published:** 2024-06-07

**Authors:** Leon Mendel McClusky, Julius Nielsen, Jørgen Schou Christiansen

**Affiliations:** 1 Anatomy Section, Department of Health & Care, Faculty of Health Sciences, UiT The Arctic University of Norway, Campus Narvik, Narvik, Norway; 2 Department of Fish and Shellfish, Greenland Institute of Natural Resources, Nuuk, Greenland; 3 Department of Arctic and Marine Biology, Faculty of Biosciences, Fisheries and Economics, UiT The Arctic University of Norway, Tromsø, Norway; Leibniz Institute on aging - Fritz Lipmann Institute (FLI), GERMANY

## Abstract

Cystic spermatogenesis in the subadult, maturing and adult Greenland shark (*Somniosus microcephalus*) displays multiple novel features, characterized early on by an unorganized internal cellular environment of the spermatocysts (anatomically discrete follicle-like units containing a single germ cell stage and its complement of co-developing Sertoli cells). These typically show polar asymmetries due to asymmetrically distributed germ and Sertoli cells. These arise from several novel cellular rearrangements at the immature pole, including fusion of a cluster of somatic cells with newly formed cysts containing only one to three spermatogonia and that already display an excess of Sertoli cells. The subadult’s germinative zone revealed an additional novelty, namely numerous previously formed somatic cell-lined rings into which spermatogonia were incorporated. A striking finding was the conspicuous rarity of the routinely discernible Sertoli mitotic figures in the hallmark cyst stage of diametric elasmobranch spermatogenesis that is known for the peak display of the latter. Scrutiny of sequentially unfolding phenomena in the linearly arranged spermatogonial generations revealed that the cellular developments at the most common type of cyst–duct transition area (comprising slender to spindle-like basophilic cells with pointed ends) were concurrent with the discreet appearance of a second dark Sertoli nucleus, a development that persisted in spermiated cysts. Spermatogenically active mature males displayed vigorous meiotic divisions. However, a scattering of their spermatid cysts also displayed shark-atypical asynchronous passage through spermiogenesis, phenomena which were exacerbated as arrested spermiogenesis in an archival collection of tissues from 13 maturing specimens. Subadult specimens revealed meiotic arrest, and foci of infiltration of leukocytes that originate from a mass of eosinophilic, granule-laden immune cells dorsally under the testis capsule. This tissue was identical to the testis-affixed bone marrow equivalent in other shark species. This tissue is likely developmentally regulated in the Greenland shark as it is absent in adults.

## Introduction

In all vertebrates, a set of cells are already set aside in the embryo for the purposes of the future production of the gametes (eggs and sperm). These specialized cells, called the primordial germ cells [1, 2] specify the development of the gonads in the embryo, including in that of an old group of vertebrates, namely sharks, skates and rays, collectively called elasmobranchs [see 3, 4 and references therein].

Given this apparent developmental urgency to sequester a distinct cell lineage that will ensure gonadogenesis (formation of the testes and ovaries) starting in the embryo, it is therefore intriguing that this dynamic organ of a vertebrate would only commence with the final maturation of gametes about156 years later, which is the case of at least the female of an extremely long-lived vertebrate and largest fish native to Arctic waters, the Greenland shark, *Somniosus microcephalus*, [5, 6]. This age-at-maturity estimate is consistent with the earlier precisely determined 0.5 cm annual growth rate in two Greenland sharks whose sex was not noted [7]. Be that as it may, the age-at-maturity for males is still unknown, but it is conservatively predicted to be at least 50 years. Although formally classified as a deep-slope shark [8], this enigmatic species displays an extensive spatial distribution in the water column, from the surface and down to at least 2.9 km and in temperatures ranging from typically from subzero to 6–7°C [9–11]. Such abiotic factors may be potentially relevant to the Greenland shark’s reproductive cycle since testicular function in ectotherms is typically attuned to these environmental cues. Indeed, no other organ in the vertebrate body displays the same plasticity in response to seasonal and exogenous cues as the gonads [12]. In vivo and in vitro experimental paradigms in better studied shark species have shown the significance of exogenous factors in testicular steroidogenesis and the seasonal waxing and waning of the protracted development of the immature germ cells (spermatogonial stem cells), spermatogonia, spermatocytes, and spermatids that transform into spermatozoa, namely the whole process of spermatogenesis (For review, [13]).

Little is known about reproductive biology of the Greenland shark or other members of the family Somniosidae that additionally contains several deep-sea species. The first focused investigation into the reproductive biology within the genus *Somniosus* concerned the Greenland sharks [14]. The latter study reported the size-at-maturity, morphological traits of different maturation stages, gross anatomical analysis of the reproductive organs, but with no histologic investigation of any of the tissues. An initial gross assessment of overall testicular status may entail a cursory glance of a histology section during which the progression of spermatogenesis is visualized as progressively enlarging lumens of the germ cell’s conventionally spherical compartments called spermatocysts. These structures, that are assembled de novo with each round of spermatogenesis, are anatomically discrete follicle-like units, that each consists of a single germ cell stage and its own set of co-developing supporting cells (Sertoli cells) that eventually become arranged around the cyst’s lumen [15, 16]. A concurrent development is the construction (at one pole of the cyst), and from the same somatic cell precursors as that of the Sertoli cells, of the germinal clone’s future exit passageway, the collecting duct, that only becomes patent upon the release (i.e., spermiation) of the spermatozoa [17, 18].

The few scattered reports on the testicular structure of deep-sea sharks indeed concerned species of the family Somniosidae, but to date only the genus *Centroscymnus* [19, 20]. Scrutiny of the accompanying photomicrographs of the immature pole of the testis of the *Centroscymnus* spp. sampled in Japanese waters [19] lacked the successive arrangement of progressively enlarging cyst lumens and instead showed uncharacteristically large and asymmetrical lumens in the newly assembled and adjacent developing cysts.

In this paper that follows up from our earlier reproductive biology study [14], we provide a first glance into the waxing and waning of spermatogenesis in immature and mature Greenland sharks, and novel observations about, among others, a similar lack of a successive arrangement of progressively enlarging cyst lumens in the spermatogenic sequence. The latter relates to several unusual findings about the expansion of the Greenland shark spermatogonial spermatocyst and its Sertoli cell numbers. Whether or not these are related to this shark’s purported exceptionally slow ≤ 1 cm annual somatic growth [7] and longevity [5], insight into some basic aspects of the cell kinetics of both its germ cells and co-developing Sertoli cells will add to the baseline of knowledge of spermatogenesis in vertebrates near the base of phylogeny [21].

## Materials and methods

### Animals

This study is therefore based on a subset of male sharks sampled as catalogued in [14] and collected as part of a collaborative effort between the ‘Old & Cold–Greenland shark project’ at the University of Copenhagen and the TUNU-Programme at UiT The Arctic University of Norway [22]. Table 1 summarizes the provisional categorization, capture date, location and morphometrics of all analyzed males. The three subadults were captured with two long lines deployed in offshore waters as part of expedition VI (in 2015) of the TUNU-Programme. Other specimens (n = 2) were acquired from unintended bycatch during the annual fish surveys of the Greenland Institute of Natural Resources. As a laboratory-based extension of our previous investigation [14], the collection of tissues in the latter study followed all relevant laws and guidelines as stipulated by expedition permit no. C-15-17 and survey license no. G15-015 granted by the Government of Greenland (Ministry of Fisheries, Hunting and Agriculture). Our investigation was also donated an archival collection of testicular and other tissues of the late professor emeritus Bjørn Berland (Norwegian Fisheries Research Institute) who sampled Greenland sharks from Norwegian commercial seal boats from 1959 to 1960.

**Table 1:** Sources, sampling periods and numbers of sharks.

	Capture date	Location	Total length (m)	Testis weight (g)	Liver weight (g)
Provisionally	Aug. 2015	Northeast Greenland	2.91	180	27.2
	Aug. 2015	Northeast Greenland	2.66	66	24.8
subadults	Aug. 2015	Northeast Greenland	2.71	200	30.4
Provisionally	Apr. 2017	Southwest Greenland	3.33	1300	78
adults	Apr. 2017	Southwest Greenland	3.04	650	N/A
Unknown status	Aug. (1959–1960)	Southeast Greenland	2.8–3.4 (n = 13)	N/A	N/A

### Histology and microscopy

The invaluable archival material includes paraffin blocks and a set of 50 histology slides of a range of dissected tissues, including testicular fragments fixed in either 70% alcohol, formol or Bouin’s fixative. Owing to the latter’s superior fixation qualities, only slides with Bouin’s fixed testicular fragments were selected for further scrutiny. Of the latter, only six slides containing sections of randomly dissected Bouin’s fixed testicular fragments were deemed useful. As these excessively thick sections (10 μm) also had numerous artefacts, a reflection of that era’s microtomy standards, one paraffin block with the largest testicular fragment was identified, melted down and the fragment re-embedded and processed for routine histology. Regarding the other freshly dissected testes: slices 5–7 mm thick were cut transversely and midway along the length of the elongated testes, fixed for 24–48 hours in 10% buffered formalin and stored in 70% ethanol until processing for routine histology and stained with hematoxylin and eosin (H&E). H&E slide preparations of formalin-fixed blue shark testes were used as positive controls.

### Microscopy

Slides were viewed and photographed with a Leica DMLS brightfield microscope fitted with a Visicam 5.0 digital camera. The arrangement of successive cyst stages across the testicular diameter resulted in a zonated appearance in any given tissue section, although the zones in the Greenland shark were not always regular.

## Results

### Spermatogenic development in the subadult testis

**Fig 1:**
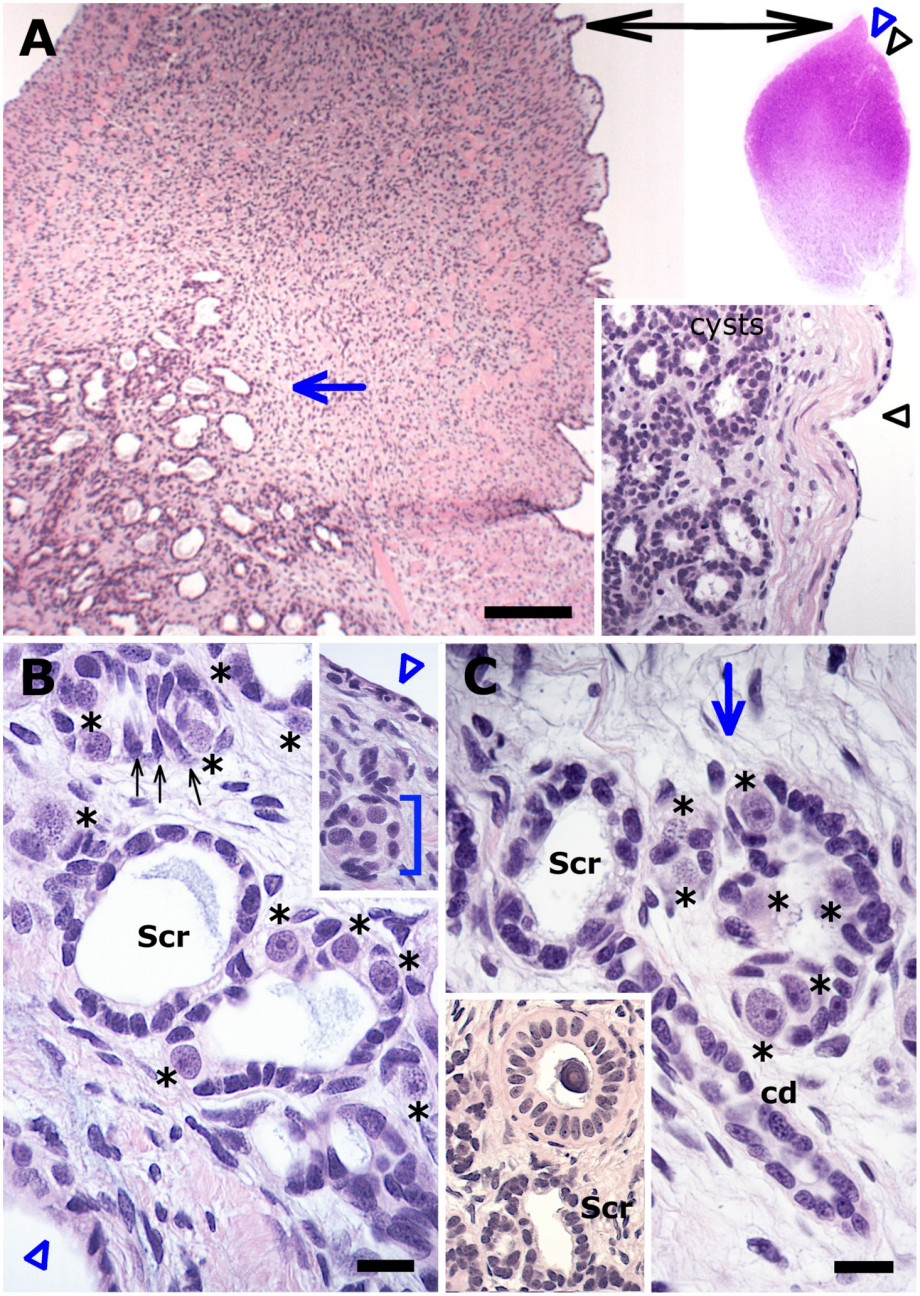
The immature pole of the subadult testis. (A) Low magnification of the germinal ridge (GR) showing numerous cell-lined spaces of various shapes and sizes medially in its dense stroma. *Insets*: a full transverse section *(upper)* of the elongated subadult testis for orientation of the GR, and approximate locations of the unorganized (blue arrowhead, blue arrow) and organized (*lower*, black arrowhead) testicular parenchyma. (B) The cell-linings of the spaces comprise either exclusively oblong somatic cells or the latter interspersed with a few large spermatogonia (asterisks), that otherwise are separately clustered together with oblong somatic cells (small black arrows) in between the spaces. *Inset*: clusters of spermatogonia (parenthesis) are also found distolaterally a short distance from the core of the GR underneath the testis capsule. (C) Another variation of the somatic cell-lined space with what clearly indicates a developing collecting duct (cd). *Inset*: The latter is distinctly different from an adjacent thick-walled intratesticular duct typically seen at the immature pole. Scr, somatic cell rings. Bar: A = 200 μm; B, C = 20 μm.

The testis of the Greenland shark falls under the category of diametric elasmobranch testes [23], as evident from a full transverse section of the elongated testis of the subadult male (Fig 1A). The outer boundaries of the organ’s immature pole were macroscopically visible as a pronounced triangular shaped germinal ridge (GR) that housed germline and somatic elements from which the testicular parenchyma is assembled (Fig 1A). At low magnification, however, the deep central region of the GR revealed numerous cell-lined spaces of various sizes and shapes among a network of ducts (Fig 1A). Closer examination revealed these spaces to be lined by either exclusively oblong somatic cells, or by somatic cells plus a few scattered large spermatogonia whose round speckled nuclei display a prominent purple nucleolus (Fig 1B). Single and various sized clusters of spermatogonia, and their accompanying somatic cells were observed among the cell-lined spaces. The cell-lined spaces and clusters of spermatogonia were also distributed distolaterally to the GR and close to the testis capsule (Fig 1B). The first signs of an organized testicular parenchyma (i.e., cysts) were observed immediately downstream in the adjacent region close to the testis capsule (Fig 1A). Another variation of the somatic cell-lined space was that with an affixed collecting duct (Fig 1C) whose assembly, by convention, is seamlessly linked to the assembly of cysts. Since duct cells and the cyst’s Sertoli cells derive from a common somatic precursor cell at the immature pole of the testis [18], all the above phenomena, including the asymmetric developing cysts (Fig 1A) seem to indicate that at least one mode of the cyst assembly in the Greenland shark involves the incorporation of spermatogonia into prior formed somatic cell rings.

**Fig 2:**
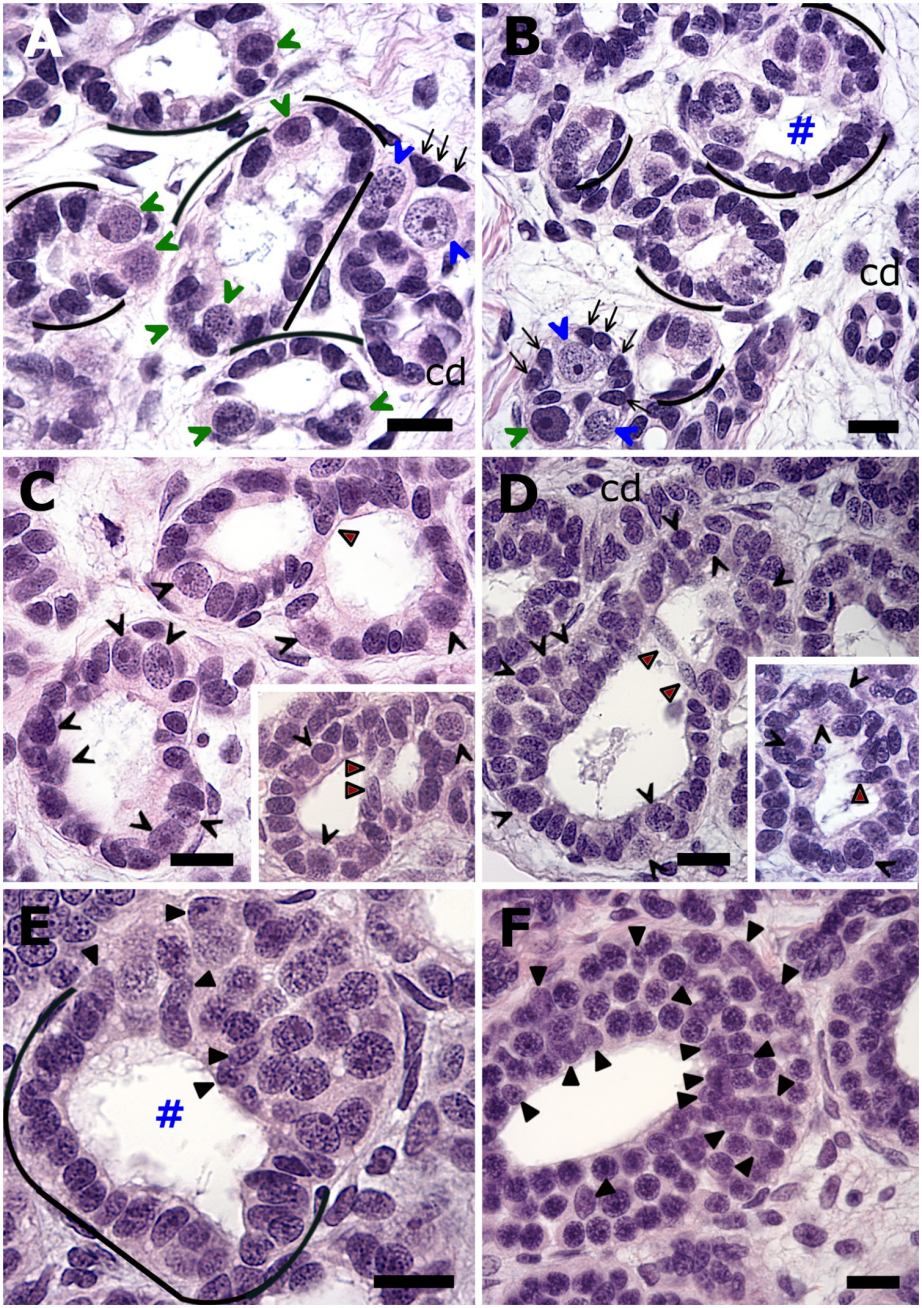
Other modes of the assembly of cysts and their fusion at the immature pole of the subadult testis. (A, B) Sometimes the clusters of primary spermatogonia reveal dark (green open arrowheads) and light speckled (blue open arrowheads) primary spermatogonial subtypes. Cysts may additionally arise from: (1) the rearrangement of aggregating oblong somatic cells (open arrows) around an increasingly discernible lumen, with the former now the new Sertoli cells (black underlined) on either side of the first two or three secondary spermatogonia, and (2) Sertoli cells that extend in a bridge-like manner towards an adjacent small cyst’s Sertoli cells giving rise to a larger cyst with a polar organization (square). (C, D) Adjoining newly formed and developing cysts, that are typically Sertoli cell-dominant, may initially share a common cyst wall (red filled arrowheads) that eventually becomes gradually disassembled due to retraction of the two or so Sertoli nuclei back into cyst periphery, culminating in cyst fusion and the “prompt” formation of a larger but asymmetrical cyst. (E) A developing cyst displaying a polar organization with bridge-like arranged Sertoli cells still distinguishable (black underlined). In contrast, Sertoli nuclei (black filled arrowheads) are less easily discerned among the opposite pole’s predominantly secondary spermatogonia, due in part, also to cross-sectioned profiles of elongated Sertoli nuclei that appear round. (F) An advanced developed ellipsoidal spermatogonial cyst showing randomly distributed Sertoli nuclei in an unevenly thick cyst epithelium. Collecting duct (cd), spermatogonia (open arrowheads, their complete numbers are shown in A, C, D). Bar = 20 μm.

The ubiquitous presence of various sized clusters of spermatogonia further distally from the GR and in the next testicular region, the germinal zone (GZ), simultaneously implied additional modes of cyst assembly. The linear arrangement of the successive developing phenomena in the diametric testis facilitated the elucidation of the assembly of cysts via other modes (Fig 2A and 2B). Some clusters of spermatogonia revealed dark and light speckled primary spermatogonia, and whose progeny were still discernible in newly assembled cysts (compare Fig 2A and 2B). The GZ, that conventionally should show successively enlarging cyst lumens, instead displayed newly formed cysts with very large lumens and other novel ways of cyst expansion. For instance, adjoining newly formed cysts with their grossly under-represented spermatogonia frequently shared a wall consisting of two or three oblong Sertoli cells, that was still manifested in what appears as a single and advanced developed pear-shaped cyst-like structure (Fig 2C and 2D). The common wall was gradually disassembled in the latter cysts in a fusion process that at some stage only revealed the wall as consisting of only cytoplasm and with the Sertoli nuclei withdrawn back into the cyst periphery. The result is the “prompt” appearance of a large, but distinctly asymmetric cyst with a polar organization due to the asymmetrically distributed germ and Sertoli cells (Fig 2D). Asymmetric cysts also arose when Sertoli cells were arranged in a bridge-like manner between two newly assembled cysts containing only one or two spermatogonia (Fig 2D). Bridge-like arranged Sertoli cells are still distinguishable in a further advanced, but lopsided-looking spermatogonial cyst (Fig 2E). The result of these different routes of cyst assembly and enlargement was seen further downstream amongst well-developed, but often ellipsoidal spermatogonial cysts that showed unevenly thick epithelia and often lacked the spatial reorganization of its cellular contents with respect to the cyst lumen (Fig 2F). Subsequent spermatogenic development could not be examined in the subadult testis because of developmental arrest at the mitosis–meiosis transition and cyst degeneration (see below).

### Spermatogenic development in the maturing and adult testes

**Fig 3:**
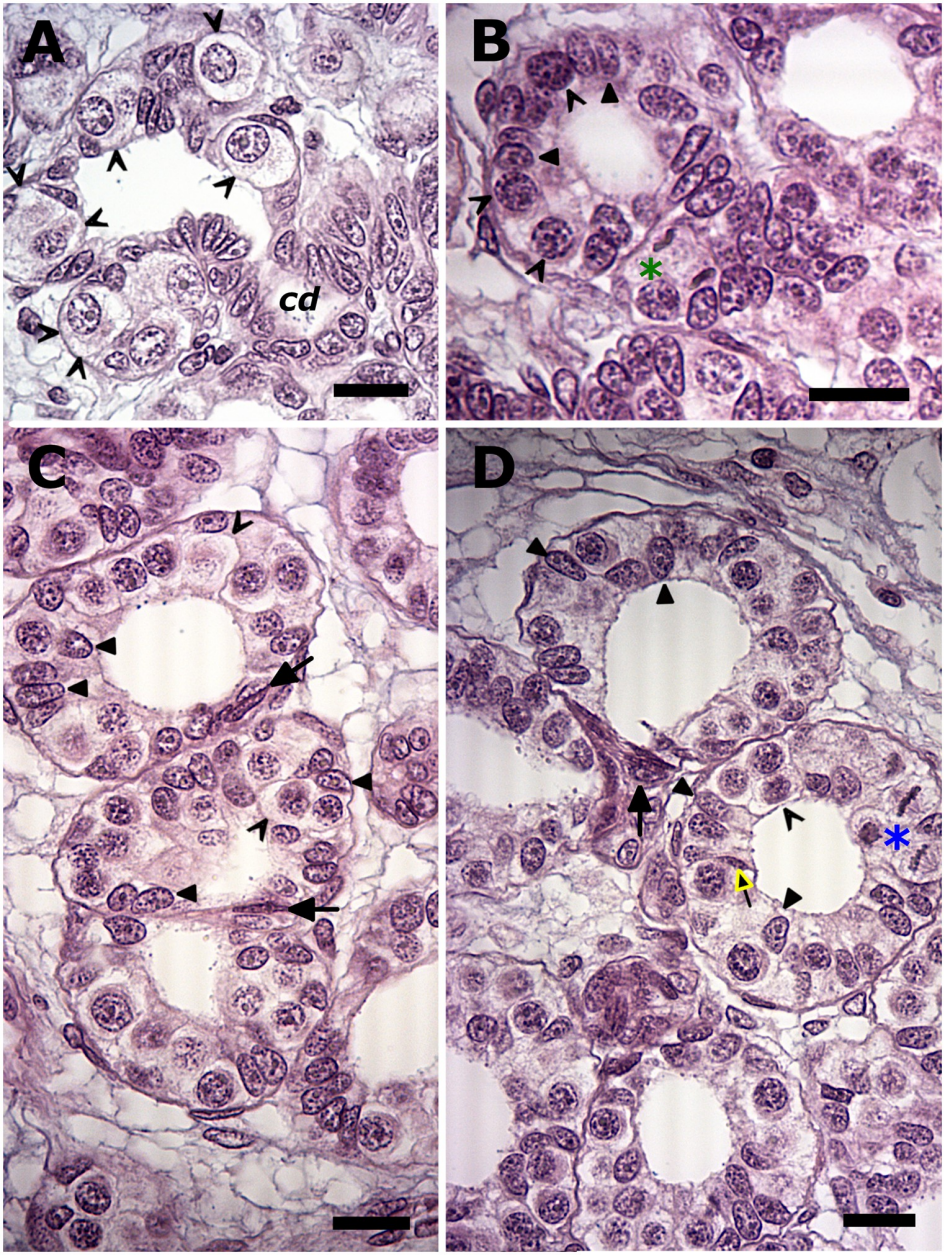
Developing spermatogonial cysts in the maturing testis (Bouins fixed tissues). (A). The appearance of spaces in the nest of primary spermatogonia (open arrowheads). Note the continuous lumen with that of presumptive collecting duct (cd). Bouins fixation accentuates visualization of the spermatogonium’s cell membrane (open arrowheads) around its voluminous cytoplasm, neither of which is discernible around the Sertoli nuclei (filled arrowheads). (B). An unevenly thick epithelium in a recently formed cyst may be due to a germ cell-deficient area at one end of the cyst, and thus a cyst with polar asymmetry. Adjacent are rarely seen metaphase figures (green asterisk) amongst somatic cells from which the collecting duct is derived. (C, D). These polar asymmetries in cysts become more overt during subsequent cyst enlargement due to the appearance of initially one or two and eventually densely packed slender or spindle-like basophilic cells (black arrows) at end of the cyst. Scrutiny of adjacent cysts reveals the appearance of a second slender or elongated dark Sertoli cell type (yellow arrow). Bouins fixation commonly accentuates the discrimination of the M-phase spermatogonia’s mitotic spindle and metaphase figures (blue asterisk) and consequently the extent of their sizes. Bar = 20 μm.

Although the GR contained many well-delineated nests of primary spermatogonia, other instances of these clustered spermatogonia revealed large spaces that were sometimes continuous with that between two rows of commonly seen small oblong somatic cells, all indicative of a presumptive collecting duct (Fig 3A). The appearance of spaces in the nest was concomitant with the observation in the adjacent area of single or doublets of primary spermatogonia with their voluminous cytoplasm, flanked by oblong somatic cells of different sizes (Fig 3A). Cysts arose from the latter and typically revealed an unevenly thick epithelium around the lumen, often due to the distinct absence of spermatogonia at one end of the cyst (Fig 3B). Since successively unfolding phenomena could be discerned in such a linearly arranged spermatogenic sequence, it was readily apparent that the polar asymmetries in newly formed cysts became more pronounced during subsequent cyst enlargement due to the appearance of initially one or two and eventually densely packed slender or spindle-like basophilic cells at the germ cell-deficient end of the cyst (Fig 3C and 3D). Scrutiny of adjoining and suitably sectioned cysts revealed the discreet appearance of a second slender or elongated dark Sertoli cell type (Fig 3D).

**Fig 4:**
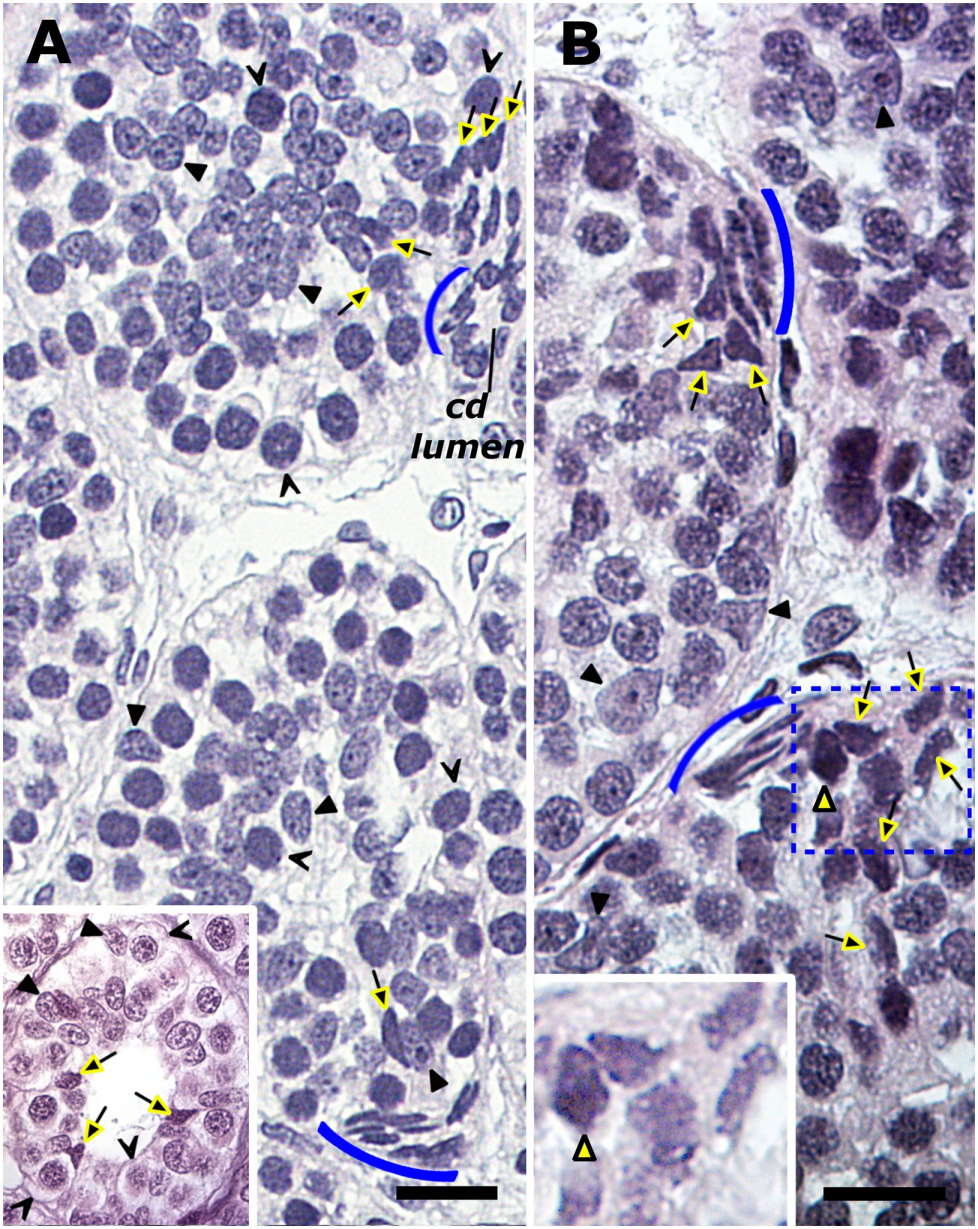
Phenomena at the most common type of cyst—Duct transition area (parentheses) in larger spermatogonial cysts in the maturing and adult testis. (A) A tangential plane of sectioning that does not include the lumen (upper) reveal slender to spindle-like basophilic cells with pointed ends (yellow arrows) among the peripherally located spermatogonia (open arrowheads). *Inset*: Bouins fixation enhances the distinction between the cyst’s original Sertoli nuclei (black filled arrowheads) and those of a second dark Sertoli cell (yellow arrows). (B) The same phenomena are observed in mature spermatogonial cysts. Different planes of sectioning also reveal a triangular shape of the second dark Sertoli cell’s nucleus. *Inset*: enhanced exposure of the boxed area to highlight a dark but bulkier looking Sertoli nucleus with an irregular profile (yellow arrowhead). cd, collecting duct. Bar = 20 μm.

The above-described polar asymmetries in developing spermatogonial cysts were related to the most common type of cyst–duct transition area, namely that comprising slender to spindle-like basophilic cells with pointed ends. Planes of sectioning that did not include the lumens of larger spermatogonial cysts offered additional insight into the dynamics of phenomena that spread along the cyst adjacent to these cyst–duct transition areas, including that of slender to spindle-like basophilic cells with pointed ends well-located among the peripherally located spermatogonia and the identification of a second dark Sertoli nucleus with pointed ends (Fig 4A). Other planes of sectioning also revealed a triangular shape of the second dark Sertoli cell’s nucleus (Fig 4B). In late-stage spermatogonial cysts, the cyst area adjacent to the opening into the collecting duct additionally showed the occasional dark but bulkier looking Sertoli nucleus with a notably irregular profile (Fig 4B).

**Fig 5:**
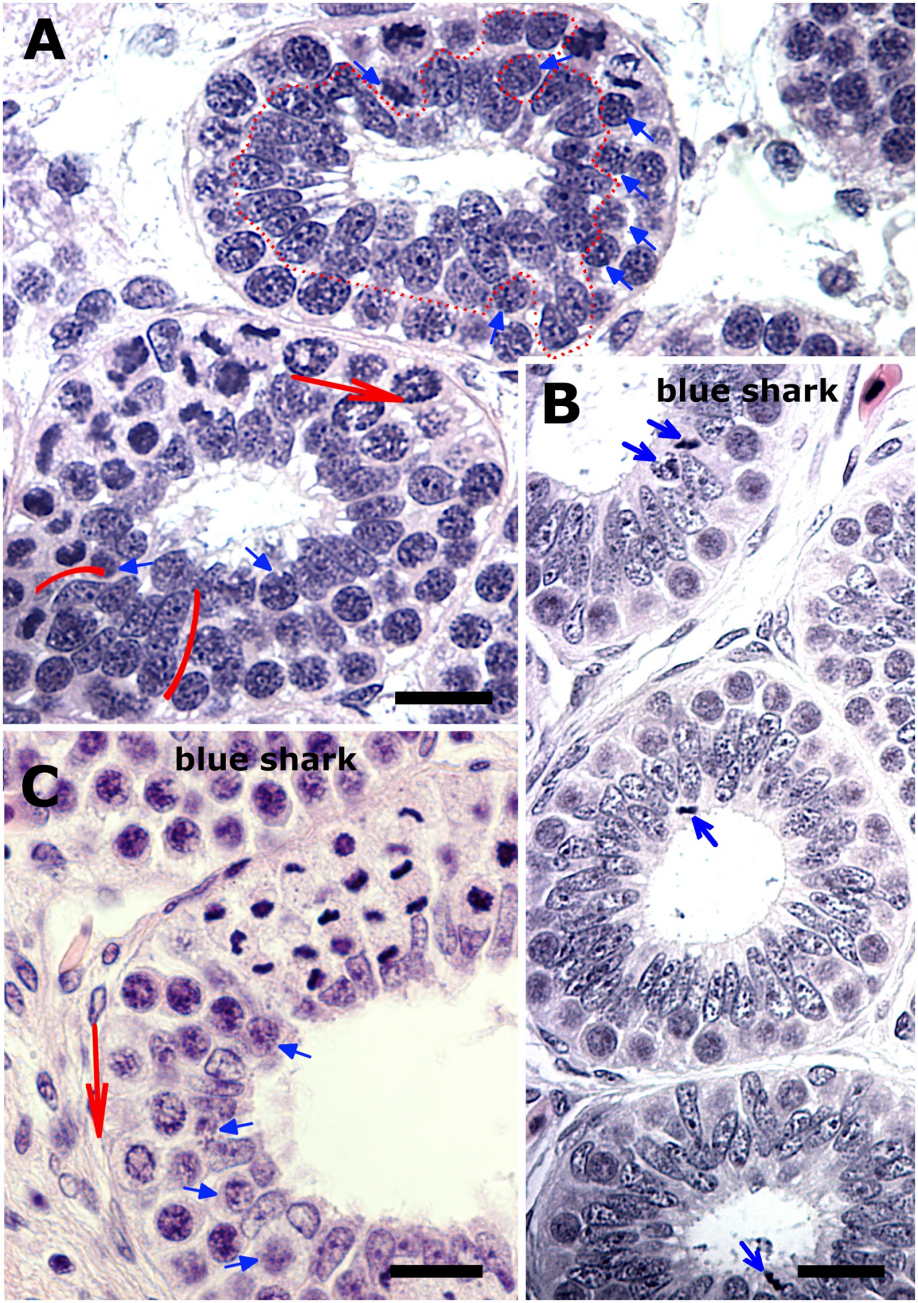
Comparable internally organized spermatogonial cyst stages in the diametric testis that are conventionally associated with frequent Sertoli cell mitotic figures seen at the luminal border. (A) Internal reorganization in the Greenland shark cysts is never complete as shown by the, at times, irregularly distributed Sertoli nuclei (encircled). Mitotic figures in such cysts are those of spermatogonia, including the odd dividing one of other spermatogonia ensconced (blue arrows) among the large bulky Sertoli nuclei. Note the next spermatogonial cyst stage’s eccentric peripheral aggregation of Sertoli nuclei (parentheses) in an odd-shaped cyst. (B, C). Positive control sections of the corresponding cyst stages in the blue shark testis showing periluminally located Sertoli cell mitotic figures (black arrows) and spermatogonial mitotic figures on the cyst periphery. The direction (red arrow) of the wave of spermatogonial mitotic activity as discerned from the increasingly dispersed chromatin in the neighboring spermatogonial nuclei. Bar = 20 μm.

Generally, in elasmobranchs, newly assembled and subsequent early developing spermatogonial cysts increase in size due to both spermatogonial and Sertoli cell mitosis (Fig 5). Whereas the sheer size of the Greenland shark’s spermatogonia was well-delineated during the display of their easily discernible mitotic figures (Fig 3D), the latter of the Sertoli cells were strikingly conspicuous by their rarity or even absence, and notably in the elasmobranch diametric testis’s first cyst stage (i.e., with periluminal Sertoli nuclei) conventionally associated with the peak display of what should be easily distinguishable Sertoli mitotic figures.

As shown in two mitotically active, successive spermatogonial generations (Fig 5A), the fringes of the dividing spermatogonia’s cytoplasm were still distinguishable regardless of whether they were on the cyst periphery or ensconced between the periluminal Sertoli nuclei. Moreover, the cyst stage conventionally and strictly associated with a wave of spermatogonial mitotic activity, displayed instead an eccentric peripheral aggregation of Sertoli nuclei in an odd-shaped cyst (Fig 5A), all of which indicate a polar asymmetric origin earlier in spermatogenesis. All of this were in stark contrast to the corresponding cyst stages in the blue shark, whose common and strictly periluminal Sertoli mitotic figures were distinct in the stage with a single layer of spermatogonia (Fig 5B), but which were completely absent in the stage with multiple peripheral layers of spermatogonia (Fig 5C).

**Fig 6:**
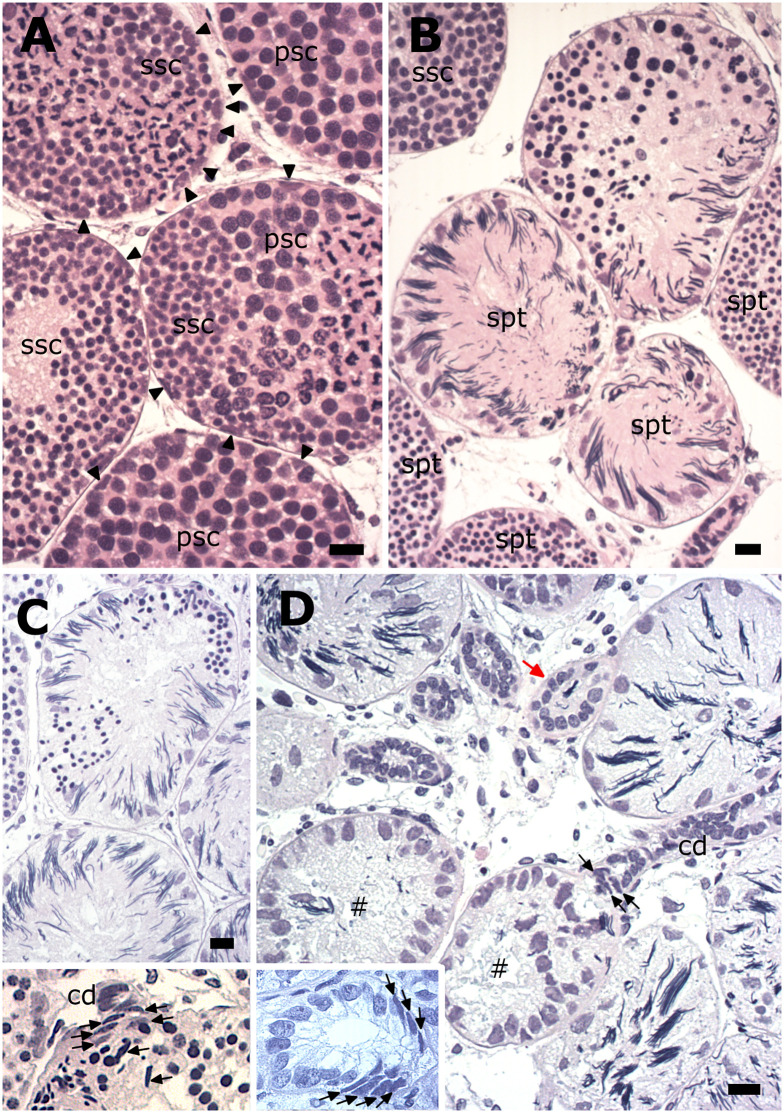
Meiotic divisions and spermiogenesis in the testis of the spermatogenically active mature Greenland shark. Both meiotic divisions (A) appear to proceed normally. However, in some spermatid cysts, spermiogenesis is notably arrested at right at its initiation (B) or is delayed leading to asynchronous development. (C). *Inset*: Appearance of a scattering of elongated dark cells with pointed ends (black filled arrows) among spermatids at the site of attachment of the collecting duct (cd). (D) Spermiogenesis and spermiation are eventually completed in most cysts as evident from the spent cysts (square) and spermatozoa in the collecting ducts (red arrow). *Inset*: a slightly tangential section of an older spent cyst with a polarized appearance. A double row of strongly basophilic elongated nuclei with pointed ends (black filled arrows) are observed at one end of the cyst. Sertoli nuclei (black filled arrowheads); primary spermatocytes (psc); secondary spermatocytes (ssc), spermatids (spt). Bar = 20 μm.

The testis of the spermatogenically active mature male displayed vigorous meiotic divisions in spermatocyte cysts (Fig 6A). However, scattered among the many synchronous, full-complement round spermatid cysts were also spermatid cysts that showed perturbed spermiogenesis. In these cysts, spermiogenesis was either arrested at the round spermatid stage, as revealed by abnormally large round spermatids (Fig 6B) or, spermiogenesis progressed to the elongation stage in one region of the cyst but was patently delayed at the round spermatid stage in another part of the cyst (Fig 6C). The site of attachment of the collecting duct to the spermatid cyst also revealed a scattering of elongated dark cells with pointed ends amongst spermatids (Fig 6C). As shown in Fig 6D, **t**he presence of spermatozoa in the collecting ducts confirmed the successful completion of spermiogenesis and spermiation. When observed, slightly tangentially sectioned older spent cysts also revealed a polar organization due to a double series of basophilic elongated nuclei with pointed ends at one end of the cyst (Fig 6D).

### Types of degenerative phenomena

**Fig 7:**
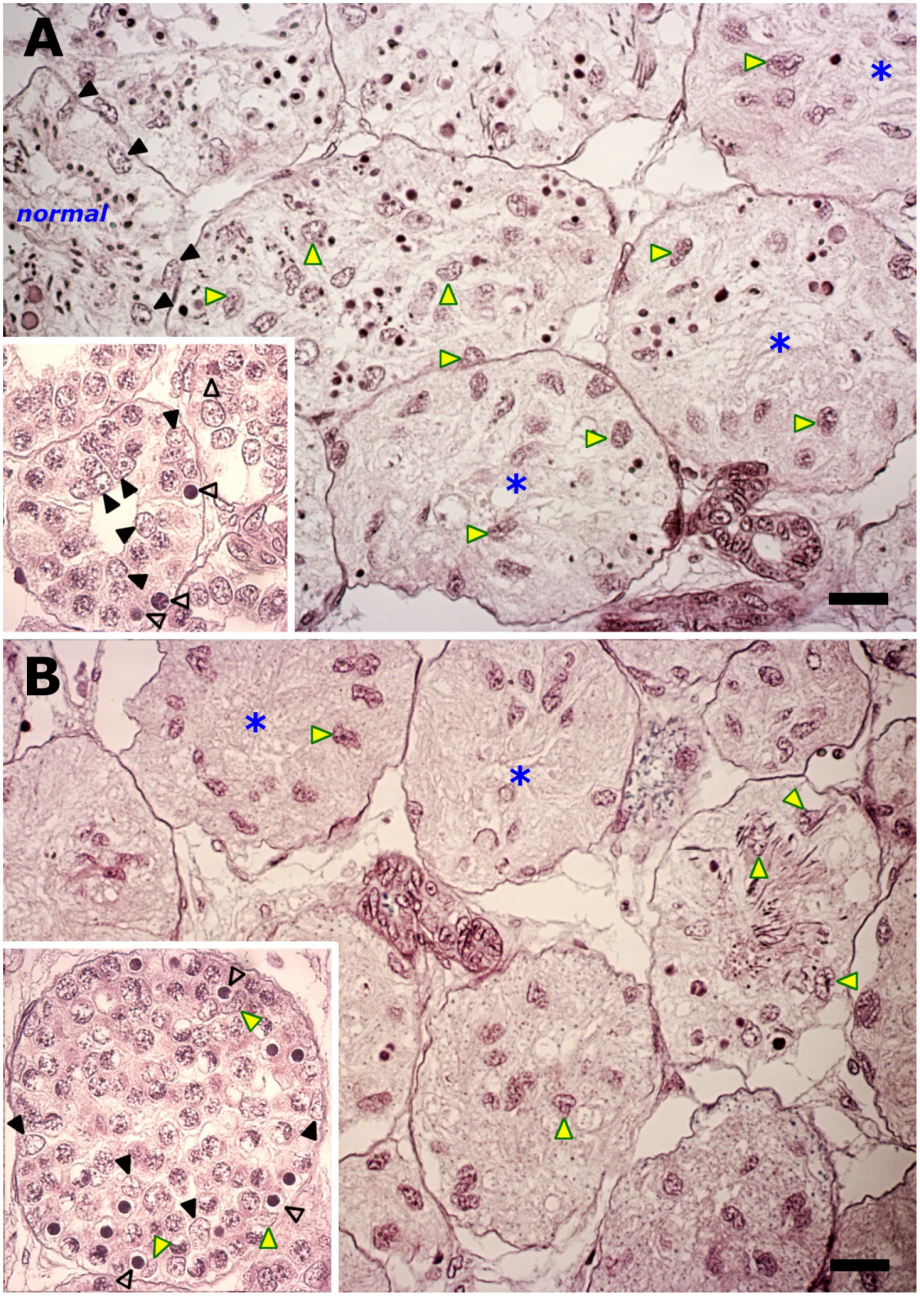
Testicular degeneration in the spermatogenically inactive male (Bouins-fixed). A prominent feature is spermatogenic arrest very early in spermiogenesis (A) that elicits heightened Sertoli phagocytic activity (yellow arrowheads) to systematically clear germinal compartment (asterisks) of the developmentally arrested, pyknotic remains of the spermatids. This a cumulative process, as evident from a zone at the mature pole of persisting phagocytically cleared cysts (B) containing only randomly distributed Sertoli nuclei (yellow arrowheads). *Insets* A, B: These testes also reveal an increasing incidence of exclusively single pyknotic germ cell deaths (open arrowheads) in successive spermatogonial generations. Note concave Sertoli nuclei (yellow arrowheads), each tightly apposed to a pyknotic body. Bar = 30 μm.

**Fig 8:**
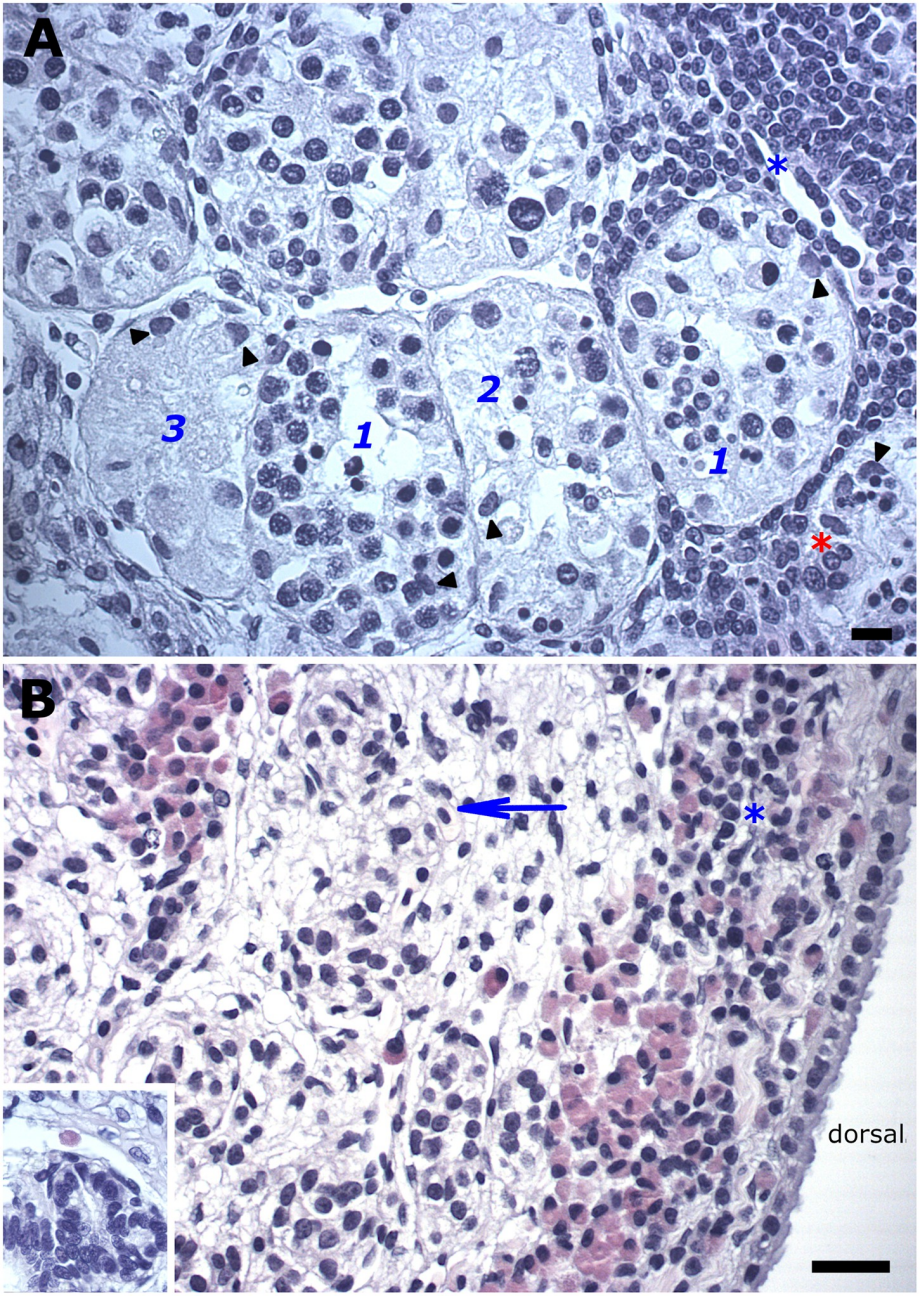
Testicular degeneration in subadult testes (formalin-fixed) manifests as arrested spermatogenesis at the primary spermatocyte stage. (A) Meiotic arrest manifests as the gradual fragmentation of the spermatocytes into pyknotic bodies (*1*), which the Sertoli cells (arrowheads) gradually clear via phagocytosis (*2→3*). However, two of the subadult testes reveal two to three well-delineated patches of leukocyte infiltration (blue asterisk) around a subgroup of these degenerating cysts followed by their invasion (red asterisk). (B). Only the testes of these two subadults reveal the source of infiltrating leukocytes, which is a compact mass of eosinophilic, granule-laden immune cells under the testis capsule. Note the discreet migration of a few of these eosinophilic immune cells in the direction (large arrow) of the numerous intratesticular ducts and degenerating cysts. *Inset*: the occasional sight of cytoplasmic shedding adjacent to involuting ducts. Bar: A = 20 μm; B = 40 μm.

Two types of stage-related testicular degeneration were commonly observed in all 13 archival collection specimens (Fig 7) and the three subadults (Fig 8). The one type of stage-related testicular degeneration entailed what at first glance resembles “empty” cysts downstream of the spermatocyte cysts and/or the arrested development of particularly round spermatid cysts. The second type of stage-related testicular degeneration in these two different sets of specimens manifested as disturbances in the developmental advance of the spermatocytes. Based on an earlier systematic analysis of similar disturbances in young and older specimens of the small-spotted catshark [24], it is best to discuss them separately.

#### Testicular tissues from archival collection

In the absence of any collected field data (e.g., testis weight, clasper characteristics), any assessment of the spermatogenic status of these specimens was therefore dependent on a retrospective analysis of their testicular histology. Compared to the spermatogenically active mature males (Fig 6), these specimens revealed clear signs of spermiogenic arrest early during spermatid elongation (Fig 7A), Making use of the linearly arranged progressive onset of spermatid degeneration seen in adjoining cysts, aborted spermiogenesis presented as anomalously large pyknotic remains of these spermatids, simultaneously as the Sertoli nuclei were increasingly randomly displaced in the cyst (Fig 7A). The significance of the latter was evident further distally in the mature pole, namely cysts with successively fewer and fewer of these anomalously large pyknotic remains of the spermatids (Fig 7A). As this was a protracted development, its conclusion presented as a thick band of “empty” cysts with only randomly distributed Sertoli nuclei that distinctly revealed a grainy appearance (Fig 7B). The distinct near absence of normal cysts containing any advanced stage of spermatid elongation confirmed the state of spermatogenic arrest. These anomalous Sertoli cell behaviors represented heightened phagocytic activity to dispose of the pyknotic remains of the arrested spermatids in these cysts. These postmeiotic degenerative phenomena were associated with a gradient of multiple and isolated pyknotic germ cell deaths seen in cysts engaged in the mitosis–meiosis transition (Fig 7A and 7B), and that tapered off in the occasional death in even in cysts engaged in the first meiotic division.

It is worth noting that the archival collection of testicular tissues displayed some animal-to-animal variation, which if properly analyzed, may aid in the understanding of spontaneously occurring perturbations of spermatogenic development in the natural environment and/or the recovery of spermatogenesis in this species. For example, the peripheral edge of the mature pole in one specimen featured only a partial row of Sertoli cell-only (i.e., completely phagocytically cleared) cysts. However, the rest of the mature pole was filled with multiple rows of cysts with primary spermatocytes, secondary spermatocytes, and with round spermatids as the last normal cyst stage.

#### Subadults

Spermatogenesis in the subadults was arrested at meiosis. This manifested as the gradual fragmentation of the primary spermatocytes into pyknotic bodies (Fig 8A). The pyknotic remains of these dead spermatocytes were gradually eliminated by the Sertoli cells in a process that culminated in the appearance of phagocytically cleared cysts (Fig 8A).

However, cross-sections of the two heaviest subadult testes (180g and 200g) additionally revealed two to three well- delineated patches of leukocyte infiltration around a subgroup of these degenerating cysts. The leukocytes invaded these cysts that still contained not yet phagocytosed primary spermatocytes and their Sertoli nuclei (Fig 8A). The leukocytes originated from a compact mass of eosinophilic, granule-laden immune cells far distally to the degenerating testicular parenchyma and just under the testis capsule (Fig 8B) in only these two specimens and not in that one with the lightest weighing testis (66g). This mass of granule-laden immune cells was identical to what is conventionally known in other elasmobranchs as the testis-associated bone marrow equivalent tissue, or epigonal organ tissue. A sparsely distributed number of these eosinophilic epigonal organ cells were also observed in the expansive connective tissue region between the compact epigonal organ tissue and the last anatomical structures distally to the degenerating cysts, namely the numerous intratesticular ducts (Fig 8B). Notably, however, these eosinophilic epigonal organ cells were never observed at the site of the meiotically arrested cysts.

## Discussion

The hierarchical arrangement of successive germinal clones in their respective cysts in a low magnification cross-section of the elasmobranch testis always manifest as progressively enlarging cyst lumens, which are virtually imperceptible at the immature pole unless viewed at higher magnification [15, 25]. Another trait of the elasmobranch testicular parenchyma is the early establishment of a hallmark internal reorganization of the cyst’s cellular contents such that exclusively Sertoli nuclei are arranged around the cyst lumen.

Here we present several novel findings in the long-lived Greenland shark that adds to the baseline of knowledge of pre-spermatogenesis and adult spermatogenesis in elasmobranchs. The spermatogonial cysts in the subadult and adult Greenland shark are not consistently spherical and eventually do not and strictly adhere to the conventional pattern of a structured internal organization. Instead, their common ellipsoidal or lobsided shapes reflect asymmetrically distributed germ and Sertoli cells. These phenomena manifest in cross-sections as differently sized cross-sectional profiles of even neighboring cysts. These observations are identical to those noted at the immature pole of another deep-sea Somniosid [19]. Our closer analysis of the pronounced GR of the Greenland shark shows multiple routes of cyst assembly. The uncharacteristically large and asymmetrical newly assembled cysts arise from several novel cellular rearrangements, including the incorporation of spermatogonia into previously formed somatic cell-lined rings (only in subadults), the fusion of the latter or clusters of somatic cells with newly formed cysts containing only one to three spermatogonia, etc. This phenomenon is also noticeable further distally in the GZ, and even between cysts that have appreciable numbers of spermatogonia.

To what extent Greenland shark’s habitat and extensive spatial distribution in the water column, from the surface and down to at least 2.9 km and in temperatures ranging from typically from subzero to 6–7°C [9–11] might underlie its sluggish somatic growth (essentially mitotic activity) which was precisely determined for two specimens (sex unknown) to be 0.5 cm per annum [7], has been much debated. The extent to which testicular cell kinetics could be impacted by such an extreme temperature regime and associated physiological traits is also not known. That which is known, however, is that no other organ in the vertebrate body displays the same plasticity in response to seasonal and exogenous cues as the gonads [12].

Against the background, one can therefore only speculate about what appear to be departures from conventional elasmobranch and vertebrate Sertoli cell biology dogma. For example, the successful development of any given germinal clone is dependent on an optimal Sertoli: germ cell ratio, with the final number of Sertoli cells in the testis setting the ceiling of sperm production, at least in mammals (see [26, 27]. Generally, in elasmobranchs, newly assembled and subsequent early developing spermatogonial cysts increase in size due to both spermatogonial and Sertoli cell mitosis. A curious finding though in the Greenland shark is the conspicuous rarity or possibly absence of what should be routinely discernible Sertoli mitotic figures in the hallmark cyst stage (i.e., with periluminal Sertoli nuclei) of diametric elasmobranch spermatogenesis that is known for the peak display of the latter, as shown previously in the blue shark [18] and again in the present study’s positive control sections. As the study of these types of phenomena typically fall outside the realm of general fisheries biology investigations into the reproductive biology of sharks, the possibility though of species differences with regards to the spermatogonial cyst stage(s) relevant for peak display of the actual Sertoli cell mitotic figures cannot be excluded. For example, mitotic figures have been observed among somatic cell precursors adjoining newly assembled cysts that in turn also subsequently fuse with them.

We therefore propose an integrative interpretation within the context of other novel findings regarding how this species may possibly ensure optimal Sertoli: germ cell ratios during each stage of spermatogenesis. The pertinent of these is the polar cellular organization of many spermatogonial cyst stages. This uncharacteristic cyst morphology arises initially from the dynamic rearrangement in the GR of the plentiful supply of somatic cell precursors, and later the fusion of clusters of somatic cells and/or somatic cell rings with newly formed spermatogonial cysts that already display a conspicuous excess of Sertoli cells. Since an optimal Sertoli: germ cell ratio is indispensable for the successful completion of the development of the germinal clone, it may be inferred that the conspicuous excess of Sertoli cells generated early on in spermatogenesis in the Greenland shark might possibly be a trade-off for its extremely low somatic mitotic rates generally [7] and/or the display of vertebrate testis’s unique plasticity in response to seasonal and exogenous cues as the gonads [12]. In other words, the polar cyst organization early on during spermatogenesis in this long-lived vertebrate Greenland may serve as a type of “reserve” or “buffer” of Sertoli cells for future multiplying spermatogonia. Support for these notions come from the observation of a partially spatially ordered, but still odd-shaped and shark-atypical spermatogonial cyst that revealed an eccentric large aggregation of Sertoli nuclei deep in the peripheral layers of spermatogonia, and moreover in the comparable differentiating spermatogonial cyst stage in the spiny dogfish [13] and blue shark where late adjustments to the germinal clone’s Sertoli cell complement never occurs. Interestingly also, no signs of cyst fusion were observed among maturing spermatogonial and all subsequent cyst stages.

As studies of this nature shed no light on causal relationships, it can only be speculated whether the inconspicuous appearance of sparse numbers of a second dark Sertoli cell type with regularity in successive spermatogonial cyst stages, spermatid cysts and even spermiated cysts are related to all the above. An advantage afforded technically during the study of the diametric shark testis concerns the *in situ* linearly arranged cysts that simultaneously disclose sequentially unfolding cellular phenomena that may be readily discernible under the light microscope. Thus, sequentially contiguous cysts immediately upstream of a particular cyst stage of interest may hold clues to earlier stages of an advanced progressed and explicit phenomenon in the cyst stage of interest located downstream. Keep in mind that abutting cysts on the same horizontal plane are at the same stage of development. It is therefore argued that the discreet appearance of a second slender or elongated dark Sertoli cell type in a cyst that abuts another cyst whose fortuitously revealed cyst–duct transition consists solely of densely packed slender basophilic cells (the overwhelmingly common type in these specimens) is not in the least happenstance (compare the sequential developments in Fig 3C and 3D). This phenomenon was first reported in the blue shark testis whose small, spatially ordered cyst’s Sertoli cells are additionally proliferatively active [18], all of which in turn raise the question about the putative functional significance of the slender to elongated dark Sertoli cell type in both these species. Quantitative analysis of the latter in the large data set of the blue shark revealed that the proportion of these small unorganized cysts with spindle-like, basophilic duct cells at the cyst opening was significantly nine-fold greater in the degenerated testicular condition than in the early recovery testicular condition of the blue shark [18]. However, the regularity of these observations in maturing and late-stage spermatogonial cysts in the Greenland shark, and not noted in the blue shark, could be more apparent than real, due to the far greater probability of sectioning through these areas in the Greenland shark’s cysts that are distinctly smaller than those typically seen in elasmobranchs. However, a significantly larger data set is needed to analyze quantitatively, and as a function of spermatogenic status, the proportions of spermatogonial cysts that display a cyst–duct transition area comprising densely packed slender basophilic cells versus cysts that display the much more infrequently observed transition area comprising elongated duct cells, as reported in the blue shark [18].

Indications are that elasmobranchs likely reveal species-specific idiosyncrasies of importance to not yet fully understood aspects of their reproductive biology and ecology. Thus, findings presented here of cysts containing early elongating and advanced elongating spermatids that like spermatogonial cysts also display a cyst–duct transition area comprising densely packed slender basophilic cells concomitant with a scattering of elongated pointed dark cells around the cyst opening and displaced amongst nearby spermatids are certainly novel as a comparable focused study of the cyst–duct transition area in the testis of *Mustelus manazo* revealed none [17]. Most intriguingly though in the Greenland shark was the distinct polar organization in especially older spent cysts (identified by their lumens totally phagocytically cleared of residual sperm) caused by a double row of elongated basophilic cells with pointed ends at one end of the cyst. Admittedly, the observation of these phenomena in spheroidal structures are crucially dependent on what essentially are fortuitous planes of sectioning of cysts viewed only in two dimensions. Due to the patently smaller sized newly spermiated cysts as recently reported [28] for the Greenland shark (about 80 μm compared to the blue shark’s 240 μm), one may reckon with a greater probability of sectioning through the cyst’s opening into the patent collecting duct. The latter study [28] that compared the protracted dismantling process of spermiated cysts in the blue shark and Greenland shark noted that, compared to newly spermiated cysts, the differentiation of the spindle-like, basophilic duct cells at the cyst opening post-spermiation becomes quite extensive in the Greenland shark, with this species-specific double row of elongated and pointed basophilic cells at one end of the older spent cyst exemplary of this cellular differentiation. Although the occasional very recently spermiated cyst in the Greenland shark may show the odd highly basophilic elongated pointed cell located slightly further away from the cyst opening along the cyst periphery, it is however typically the older spent cyst of only the Greenland shark that readily reveals a deep internally located highly basophilic elongated to ovoid nucleus of second Sertoli cell type [28] that is identical to that seen in its spermatogonial cysts (the present study).

The postmeiotic degenerative phenomena seen in the spermatogenically inactive males are in principle consistent with that seen in at least one other oceanic shark species, i.e., the blue shark [29]. As in the blue shark, the gradient of postmeiotic degeneration in the Greenland shark was associated with elevated germ cell deaths in cysts engaged in the mitosis–meiosis transition, the latter also known to occur in seasonally migrating spiny dogfish [13]. However, the gradient of spermatogonial apoptotic deaths at the mitosis–meiosis transition in the blue shark and spiny dogfish [13, 29], manifests clearly as the fusion of dying neighboring clone members in the cyst, a development that was never seen in either the Greenland shark’s significantly affected spermatogonial cysts (see Fig 7) or even in the occasional incidence of death in spermatocyte cysts, which again and interestingly never display cell death in either the spiny dogfish and blue shark.

The absence of any accompanying field-collected morphometric data of this large-bodied species renders premature any assumption of the spermatogenic status of 13 archival collection specimens based solely on the histologic examination of the testis. A major consideration is the animal-to-animal variation seen among these 13 specimens that nevertheless consistently shared the following histological traits, namely a spermatogenic sequence (1) in which the distinctly and uncharacteristically sub sized spermatogonial cysts (see Fig 7A) were loosely, and not densely packed in the spermatogonial region, (2) that completely lacked any cysts containing advanced elongating spermatids nor old spent (spermiated) cysts, and (3) with either a thick band or one row of completely phagocytically cleared cysts that were consistently the last cyst structures at the mature pole. Moreover, the spermatid cyst stage targeted for phagocytic clearance by the Sertoli cells was regularly the very earliest stage of spermatid elongation. The total lengths (2.8–3.4 m) of these 13 specimens fall within the range of mid-Canadian Arctic specimens (3.11 m and 3.71 m) that were classified as sexually immature [30, 31]. Several pieces of data clearly indicate that the 13 specimens were not subadults. Firstly, meiotic arrest at the primary spermatocyte stage in the subadults entailed foci of leukocyte infiltration from the adjacent small mass of epigonal organ tissue in identical manner to meiotic arrest in juvenile spiny dogfish (personal observations). Secondly, the aborted development early in spermiogenesis in the 13 specimens elicited a phagocytic response, but which was strictly cyst-intrinsic (i.e., invoking Sertoli phagocytic clearing activities), and which strikingly resembles that observed in summer-mating blue sharks [29]. Moreover, all 13 specimens showed evidence of meiotic divisions, with one specimen even revealing normal large round spermatids cysts as the penultimate cyst type at the mature pole, all of which suggest that these sharks were likely approaching maturity. The data are unfortunately insufficient to assist towards any inferences about whether the latter developments concerned novice or experienced males.

An interesting finding in only two of the subadults (those with the heaviest testes) concerns two to three well-delineated foci of aggregating leukocytes around a subgroup of these degenerating cysts. The leukocytes, that originate from a compact mass of eosinophilic, granule-laden immune cells dorsally just under the testis capsule, invaded these cysts that still contained a few not yet phagocytized spermatocytes. In several shark species, including the blue shark [29] and other species [32], these distinctive eosinophilic, granule-laden immune cells are the dominant constitutive elements of the massive testis-affixed bone marrow equivalent (i.e., epigonal organ). Fange and Mattison [32], however, reported that the Greenland shark lacked an epigonal organ or only had traces of it. Although not at all observed as a conspicuous mass in the adults examined here, the findings presented here for two of the subadults conclusively show the involvement of discrete foci of epigonal-derived non-eosinophilic leukocytes in the phagocytic clearing up of the testicular parenchyma in the aftermath of meiotic arrest. Mindful that these are histologic observations in wild-caught animals, it is nevertheless still noteworthy that only the testes of these two subadults showed the discreet migration of a sparse number these eosinophilic epigonal cells away from the epigonal organ tissue and eventually among both intact and involuting intratesticular ducts, but never anywhere close to the meiotically arrested cysts. In contrast, the massive epigonal organ of similarly wild-caught adult blue sharks never releases any of its constitutive eosinophilic epigonal cells irrespective of the animal’s spermatogenic status [33] and is histologically unresponsive to cestode parasites even attached to the epigonal organ’s surface [34].

Taken together, these findings presented here suggest that this epigonal tissue is transient and likely developmentally regulated in the Greenland shark, as it was lacking in specifically the subadult testis with the lightest weight and with no traces of it any of the adults examined. This broadly resembles the findings of the developmentally related appearance of the epigonal organ in two other deep-sea Somniosids [19].
